# Effects of the narrative elaboration technique and open-ended rapport on accuracy of children’s recall according to age

**DOI:** 10.3389/fpsyg.2024.1298759

**Published:** 2025-01-09

**Authors:** Seungjin Lee, Minkyeong Shin

**Affiliations:** ^1^Konkuk University, Seoul, Republic of Korea; ^2^Other, Portland, OR, United States

**Keywords:** narrative elaboration technique, open-ended rapport building, child memory, age difference, investigative interview

## Abstract

**Introduction:**

This study explored the impacts of the narrative elaboration technique (NET) and open-ended rapport building on younger (*n* = 30, ages 5–6) and older (*n* = 30, ages 7–8) children’s free recall and suggestibility.

**Methods:**

Children were randomly assigned to either a NET condition or an open-ended rapport condition after engaging in a photo-taking play session with an experimenter. Then, a novel interviewer asked them about the play session. The effects of the experimental conditions on children’s free recall and suggestibility were examined according to children’s age groups.

**Results:**

Results revealed that open-ended rapport played a more significant positive role in young children’s free recall and suggestibility when compared to NET. No significant differences were observed in the effects of NET and open-ended rapport on older children’s memory performance.

**Discussion:**

Practical guidance is provided in terms of prioritizing open-ended rapport over NET to maximize young children’s spontaneous and accurate recall.

## 1 Introduction

Research has shown that young children can accurately report on their personal experiences, such as stressful events or repeated harm over a long period (Baker-Ward et al., 2015; Fivush, 2011). Yet younger children (i.e., those aged 6 or below with immature cognitive and language development) tend to face challenges when asked to recount their experiences in detail (Lamb et al., 2000; Sternberg et al., 2001). They are especially susceptible to interviewers’ suggestive questions, which can lead to incomplete and inaccurate reporting (Lyon, 2014; Saywitz and Camparo, 1998, 2014). Along with cognitive and verbal limitations, various factors can affect younger children’s recall: characteristics of the experienced events (Pathman et al., 2013); the types of questions posed (Mehrani and Peterson, 2015; Rocha et al., 2013); individual differences (Klemfuss and Olaguez, 2020); and supplementary interview tools (Otgaar et al., 2016). Memory recall accuracy tends to be consistently higher when a child voluntarily answers the interviewer’s open-ended questions (Powell et al., 2022). However, younger children’s spontaneous recall generally lacks richness and specificity (Levine et al., 2002). A child’s testimony based on open-ended questions may thus be insufficient for judicial adjudication if the child is the lone information source on the nature of the perpetrator and perceived negligence (Duron, 2018). Discrepancies in police officers’ investigative competencies can also produce evidence of variable quality and validity (Lamb, 2016; Powell et al., 2009). Scholars and practitioners have expressed growing interest in investigative interview procedures or complementary guidelines that will minimize the contamination of younger children’s memories and maximize reports’ accuracy and specificity (Brown and Lamb, 2019).

Effective investigative interview practices protect children’s rights by eliciting credible victim accounts and help prevent adults from being falsely accused based on misstatements (Brubacher et al., 2020; Denne et al., 2020). Researchers have recommended numerous options when interviewing children to facilitate recollection (Fisher and Schreiber, 2017). Debate persists around early-stage investigative interview techniques that may reduce a child’s susceptibility to suggestive questions and increase the accuracy of the child’s testimony (Meissner, 2021). Open-ended rapport building and the narrative elaboration technique (NET) have been suggested as alternative investigative strategies that could remedy the shortcomings of previous techniques. With open-ended rapport, the investigator aims to establish a connection with the child using open-ended questions; the child can then more easily respond to the investigator’s inquiries autonomously and confidently while resisting suggestive ones (Roberts et al., 2004). NET focuses on improving children’s narrative skills by using content unrelated to the events of interest (Camparo et al., 2001). Younger children largely lack an understanding of which details to report and how to communicate in a situationally appropriate fashion even if they remember the events they have experienced (Saywitz and Snyder, 1996). NET helps children voluntarily and specifically describe core event-related information during investigative interviews by providing children opportunities to learn about the types of details that judicial authorities need (Saywitz and Camparo, 2014). However, to date, no study appears to have compared these two methods’ effectiveness among children, especially those aged 6 or younger.

### 1.1 Open-ended rapport building

Open-ended rapport building refers in this context to a strategy where the interviewer attempts to establish rapport with a child by posing open-ended questions. When working with a child who is reluctant to provide critical statements, it may be beneficial for the interviewer to display a warm, supportive attitude early in the interview to prevent poor dynamics caused by the child’s anxiety (Ponizovsky-Bergelson et al., 2019; Saywitz et al., 2019). The interviewer can ask open-ended questions (e.g., “I want to know you well. Can you tell me a little more about you?” and “Talk more about what you said before.”) to build open-ended rapport (Price et al., 2016; Roberts et al., 2004). The revised National Institute of Child Health and Development (NICHD) Child Investigative Interview Protocol stresses efficient rapport building (Blasbalg et al., 2021; Hershkowitz et al., 2014). Open-ended rapport has been specifically incorporated into this protocol (La Rooy et al., 2015). In addition to suggesting ways to establish and maintain decent rapport with a child (e.g., calling the child by name, showing interest in their story), the protocol recommends giving the child opportunities to spontaneously recall recent experiences using neutral content that is personally meaningful at the beginning of the interview (Lamb et al., 2018). The protocol further underlines the importance of providing encouragement and positive feedback about the child’s effort and willingness to share their experiences (Brubacher et al., 2019).

Research on open-ended rapport has demonstrated this technique’s advantages for investigative interviews. For example, Sternberg et al. (1997) compared the effects of open-ended questions (e.g., “Tell me about yourself.”; open-ended rapport condition) and direct and focused questions (e.g., “How old are you?”; direct rapport-building condition) on children’s memory recall when establishing rapport with children who were crime victims. In that study, children in the open-ended question condition provided roughly 2.5 times more event-related details than children in the direct-question condition. Roberts et al. (2004) similarly compared recall details and accuracy among 3- to 9-year-olds assigned to either an open-ended rapport condition or a direct rapport-building condition. Findings indicated that the recall accuracy of children in the open-ended rapport building condition was higher than for children in the direct rapport-building condition.

Scholars have argued that open-ended rapport enhances children’s recall for several reasons. According to Fisher and Geiselman (1992), open-ended rapport building shifts control of the interview from the interviewer to the child interviewee. This transition can be particularly effective when using open-ended questions that give the child the impression that they are the expert, not the interviewer. Open-ended questions enable children to think about and choose what information to report. The memory recall theory from cognitive psychology posits that interviewers who quantitatively and qualitatively improve witnesses’ and victims’ recollection conduct interviews centered on children and give them sufficient time to think and respond. This interview strategy also signals that the child is more knowledgeable than the interviewer and allows the child to direct the conversation. As a result, children develop a sense of responsibility as information providers (Arterberry, 2022). When children can confidently respond autonomously, they are more likely to resist the interviewer’s misinformation and to show increased motivation for voluntary recall (Hritz et al., 2015).

Furthermore, researchers have asserted that cultivating open-ended rapport affords children opportunities to practice using appropriate recall tactics (Roberts and Blades, 1999). Open-ended rapport also enables children to practice describing themselves and to learn how to volitionally provide information. Children can further enrich their statements by adding details to supplement what they have already stated. Conversely, direct questions make it difficult to transfer control by bringing up interviewer-chosen topics that are unrelated to the child’s previous statements. Under such circumstances, children may answer more passively and be more susceptible to suggestibility; they feel less control over the type of information they can share and are less confident about the information they have. Therefore, open-ended rapport might be more important for younger children who are developmentally immature than for older children. It would hence be helpful to examine the effectiveness of open-ended rapport on children’s memory performance depending on their age.

### 1.2 Narrative elaboration technique

NET is a method of providing children with communication training before investigative interviews or improving children’s narrative abilities by practicing responses to open-ended questions (Lamb and Brown, 2006; Saywitz and Camparo, 2014; Saywitz and Snyder, 1996; Saywitz et al., 1996). NET helps children adapt to unfamiliar interview situations and interview tasks without contaminating statements. It aids in children’s recall by offering neutral retrieval cues that are necessary to organize memories while minimizing communication errors and suggestibility. Young children tend to lack the knowledge, experience, and communication skills suitable for investigative interviews (Melinder et al., 2021). Considering that training and practice tests can enhance one’s future performance (Polack and Miller, 2022), NET could improve memory recall in children who have not encountered investigative interview–type questions that require voluntary statements (Brown and Pipe, 2003a).

NET prompts the retrieval of descriptive information by category: four picture cards are presented that, respectively, depict people, emotions, behaviors, and environments related to specific events (Brown and Pipe, 2003b). NET consists of three phases: the preliminary phase, the core interview phase, and the closing phase (Saywitz and Camparo, 2014). The preliminary stage consists of the interviewer introducing themselves, building rapport, and giving guidance on basic rules. If required, the interviewer performs communication exercises to prompt the child’s spontaneous narrative and reduce suggestibility. The core interview stage, which is the most important for investigative interviews, comprises three tasks: free recall, cue elaboration, and direct questioning. Free recall is the process by which children produce spontaneous narratives. For instance, children’s recollection can be encouraged by posing open-ended questions such as “What happened?” and then adding one or two open-ended questions to obtain additional information (e.g., “Can you tell me more?”; “What happened next?”). Cue elaboration refers to narrative elaboration, in which the interviewer offers visual or verbal clues to help the child disclose more details about people, places, actions, conversations, and emotional states related to the incident. These clues not only prompt information retrieval but also maintain a focus on key people, places, actions, conversations, and emotional states involved in the case (as is obligatory for investigative interviews). For example, questions about people may concern who was present during an event and how they looked; location-based questions center on where the event took place and how the location appeared; and action questions address where the people present during the event were and what they did. For questions about conversations and emotional states, the interviewee is asked to share more about what they themselves said and felt. Interviewers can use the visual cues that, respectively, represent a person, place, action, conversation, and emotional state. The child must be given a description of each card in the preliminary stage and practice using them. Once the child understands the cards’ meanings and provides voluntary statements via free recall, the interviewer can display the cards one by one during the cue elaboration process and ask if the child has more to say about each category. Cue elaboration occurs between free recall and direct questioning to help the child elaborate disclosed information. The amount of additional insight acquired through cue elaboration can range from at least twice to as much as eight times of that gathered from a general interview, yet the number of errors does not increase. As the interviewer obtains more details about the event, interpretation bias can decrease along with the child’s suggestibility and compliance (Maras and Bowler, 2014). Lastly, during direct questioning, the interviewer asks follow-up questions to clarify ambiguous or inconsistent information from the child’s key statements during free recall and cue elaboration. At this time, the interviewer should ask non-leading questions based on what the child has said. The closing stage concludes the interview: the interviewer responds to the child’s questions and informs the child of next steps. If the child seems stressed, the interviewer provides appropriate coping strategies to help the child regain emotional stability.

Several studies have indicated that children receiving NET training exhibited increased memory performance without a significant increase in erroneous information when compared with those who did not have such training (Bowen and Howie, 2002; Peterson et al., 2013). Parallel results were found in children who were in preschool (Bowen and Howie, 2002; Dorado and Saywitz, 2001) and elementary school (Brown and Pipe, 2003a, 2003b; Camparo et al., 2001; Nathanson et al., 2007; Saywitz and Snyder, 1996; Saywitz et al., 1996). The types of information children report can vary, though. Children with (vs. without) NET training have been found to offer more information during investigative interviews about the “person” who was engaged in the case and whom the children considered to be important (Brown and Pipe, 2003b). Peterson et al. (2013) found that children in the NET condition reported their thoughts, emotions, and experience-based descriptions (e.g., setting, people, events) in greater depth than children in the control group.

### 1.3 Research questions

Crime investigations involving child victims come with inherent challenges. Younger children’s developmental immaturity and brief attention span restrict the period for which these participants can focus during an investigative interview (Saywitz and Camparo, 2009). Investigators may therefore face challenges obtaining complete, accurate statements from children within the allotted time (Bull, 2014; Cleary, 2017). NET and open-ended rapport have been deemed useful in enhancing children’s recall of their experiences and in diminishing interviewers’ suggestibility. However, it can be cumbersome to use both interview strategies to improve children’s memory before an investigative interview considering obstacles that limit investigators’ time. It is accordingly important to identify which interview strategy may be more time-efficient and effective in promoting children’s memory performance, especially for interviewees who are 6 years old or younger.

In our study, we compared the impacts of NET and open-ended rapport on children’s free recall and suggestibility, considering children’s age. We formulated a directional hypothesis and tested an exploratory research question:

1. We expected older children to have better memory performance and to be less suggestible than younger children.
2. We compared the efficacy of NET training and open-ended rapport building without an *a priori* hypothesis. We also explored whether these strategies’ efficacy would be dependent on children’s age range.

## 2 Materials and methods

### 2.1 Participants

Sixty-two children between 5 and 8 years old who resided in the metropolitan area of Seoul, South Korea were recruited for this study. The research team initially reached out to potential participants’ parents based on contact information the team had collected through longitudinal research. The child participants were mainly recruited from preschools, kindergartens, and art academies in Seoul. Two children were excluded from the final analysis because their emotional state or lack of attention resulted in incomplete interviews. Therefore, data from 60 children (30 boys, 30 girls) were analyzed. The participants were divided into two groups based on age: 5–6 years old (*M* = 69.47 months, *SD* = 6.16, range: 61–78) and 7–8 years old (*M* = 94.83 months, *SD* = 7.69, range: 84–106). Each age group had 30 participants.

Power analysis was conducted to confirm the effect size for the influences of two experimental conditions (i.e., open-ended rapport building and NET training). Calculations for the proposed statistical model were performed in G*Power 3.1 software (Faul et al., 2007). A sample size of 60 or more was deemed adequate to meet our objectives based on a two-way analysis of variance (two-way ANOVA; power = 0.85, *α* = 0.05, small effect size of 0.10–0.20). The target sample size was 60. We adopted a random sampling method but recruited participants in a way that allowed for a similar gender distribution in each age group. No sampling restrictions were imposed apart from age and gender.

### 2.2 Procedure

One member of the research team, who holds a master’s degree in counseling psychology and has extensive experience interviewing children, described the study purpose and process to the parents and then described these aspects to the children using age-appropriate language. The researcher answered any questions the family had. The experiment was conducted only after parents and children fully understood the research and expressed their intentions to participate voluntarily. Parents of participating children were provided with a copy of the consent form to review and keep. The study consent and permission forms were in Korean.

After parental consent and child assent were obtained, children were told they would enter a playroom with an experimenter and complete various activities. Participants were guided to an area decorated as a playroom and engaged in a photo-taking play session (adopted from Lee and Kim, 2023). Children had natural physical contact with the experimenter throughout the session. No tasks that might induce negative emotions (e.g., anxiety, withdrawal, discomfort) were included. After the play session, the children took a 5-min break; this short reprieve was crucial considering children’s limited attention span. Afterwards, children were randomly assigned to one of two experimental conditions, either an open-ended rapport building condition or a NET training condition. There was no control group. Participants were allocated in a way that allowed for a similar gender distribution per group. Tables 1, 2 list the number of children by age and experimental condition.

**Table 1:** Means, standard deviations, and two-way ANOVA for children’s free recall.

Variable	Descriptive statistics			ANOVA		
	*n*	*M*	*SD*	*F*(1,56)	*p*	Partial η^2^
Main effect						
Age				184.72	<0.001	0.77
Young	30	0.17	0.08			
Old	30	0.43	0.08			
Condition				16.24	<0.001	0.23
NET	30	0.26	0.17			
Open-ended rapport	30	0.34	0.14			
Interaction effect				2.63	0.11	0.05
Young, NET	15	0.11	0.08			
Young, Open-ended rapport	15	0.22	0.04			
Old, NET	15	0.40	0.07			
Old, Open-ended rapport	15	0.45	0.09			

*N = 60. ANOVA, analysis of variance; Young = children who were 5–6 years old; Old, children who were 7–8 years old; Condition, experimental condition; NET, NET condition; Open-ended rapport, open-ended rapport condition; Young, NET, younger children in the NET condition; Young, Open-ended rapport, younger children in the open-ended rapport condition; Old, NET, older children in the NET condition; Old, Open-ended rapport, older children in the open-ended rapport condition.*

**Table 2:** Means, standard deviations, and two-way ANOVA for children’s suggestibility.

Variable	Descriptive statistics			ANOVA		
	*n*	*M*	*SD*	*F*(1,56)	*p*	Partial η^2^
Main effect						
Age				33.65	<0.001	0.38
Young	30	0.14	0.09			
Old	30	0.04	0.05			
Condition				11.95	0.001	0.18
NET	30	0.12	0.10			
Open-ended rapport	30	0.06	0.06			
Interaction effect				4.87	0.03	0.08
Young, NET	15	0.19	0.09			
Young, Open-ended rapport	15	0.09	0.07			
Old, NET	15	0.05	0.05			
Old, Open-ended rapport	15	0.03	0.04			

*N = 60. ANOVA, analysis of variance; Young, children who were 5–6 years old; Old, children who were 7–8 years old; Condition, experimental condition; NET, NET condition; Open-ended rapport, open-ended rapport condition; Young, NET, younger children in the NET condition; Young, Open-ended rapport, younger children in the open-ended rapport condition; Old, NET, older children in the NET condition; Old, Open-ended rapport, older children in the open-ended rapport condition.*

Depending on the assigned experimental condition, children were provided with either open-ended rapport building or NET training. They were then assessed on their recall of the photo-taking play session by an interviewer who was blind to the conditions that the children experienced. Memory interviews lasted 15–20 min on average. Active encouragement and positive feedback were continuously provided to persuade children to think of the entire process as entertainment rather than an evaluation.

### 2.3 Open-ended rapport building

Established procedures (e.g., Roberts et al., 2004) were used in the open-ended rapport building condition. For each interview, the experimenter first introduced themselves to the child assigned to this condition. Then, the experimenter asked open-ended questions (e.g., “I want to know you well. Can you tell me a little more about you?”) about neutral but personally meaningful topics to build rapport and enable the child to practice talking about these matters. Topics could concern the child, family, school, or a recent special occasion. Each child was first presented with a prompt (e.g., “Tell me about yourself.”; “Tell me about your family.”) with the interviewer recording the child’s answers. If a child could not respond regarding the target topic, semi-open-ended questions were asked to help elicit answers (e.g., “How old are you?”; “What is your favorite food?”). The free memory recall phase began once the child provided information (e.g., “Can you tell me more about [something the child mentioned]?”). The interviewer used semi-open-ended questions (e.g., “So what happened?”; “Can you tell me more about it?”) or cued invitations (e.g., “You talked about a person/object/behavior. Can you tell me more about it?”) to obtain specifics and facilitate the child’s spontaneous recall. Once the child’s voluntary description (as acquired through open-ended questions) ceased to reveal new information, the interviewer proceeded with “wh”-type questions on topics the child had mentioned previously. For instance, if a child discussed their birthday cake, the interviewer asked questions such as “What color was the birthday cake?.” The interviewer posed specific questions if important information had still been omitted by the end of the interview. These lines of questioning mainly followed a “yes/no” format. The questions were often focused on obtaining more new information about what the child had described in the open-ended recall stage. This way, the child could practice detailing their experience, which familiarized them with the type and amount of information that should be shared with the experimenter. It also increased the child’s confidence and responsibility as an information provider because the child was treated as being more knowledgeable than the experimenter and was given control over the conversation and its information. After all questions had been answered, a new experimenter asked the child to evaluate rapport quality. Scores ranged from −10 to +10, with −10 being very uncomfortable, 0 being normal, and +10 being very comfortable. The child was then thanked for their participation. A short closing statement ended the interview.

### 2.4 Narrative elaboration technique

Established NET procedures were adopted from Brown and Pipe’s (2003a, 2003b) study. Children in the NET training condition first watched a short video with the experimenter, which consisted of behaviors related to children’s hygiene (e.g., washing hands, brushing teeth, washing face) and routine checks experienced in a hospital (e.g., measuring height and weight). After watching this video, the experimenter conveyed to the child the importance of reporting accurate information without guessing or fabricating events’ content. The experimenter next introduced four picture cards, respectively, covering participants, actions, settings, and conversations and explained what each image represented (e.g., “This card symbolizes ‘participants,’ which means describing the appearance or characteristics of the people who appeared in the event in detail.”). Each card was used to recall what the child saw in the video. Feedback was provided concerning the accuracy of the reported content. For example, if a child’s memory was false (e.g., the child reported having experienced something that they had not), the child was given feedback about the correct answer. If the child did not respond, the card category was noted to be unmentioned. Children were reminded of the meaning of each card on which they were being trained before moving on to the next. After training with all four cards, the child was offered an opportunity to spontaneously recall events (e.g., the child’s experience that morning) whose accuracy the experimenter could not determine. By presenting the cards one by one, the experimenter could verify whether relevant information was recalled and whether the child understood the card’s meaning correctly. If the child’s report was inaccurate, the experimenter corrected the child. The training ended with a review of the cards’ meanings.

## 3 Measures

### 3.1 Memory performance

A new interviewer who did not participate in the play session and was blind to the children’s experimental conditions evaluated participants’ memory recall. The interview pertained to the initial play session, which involved taking pictures with the experimenter. Objects and actions that appeared during play constituted items for the children to remember.

At the beginning of the memory interview, the interviewer introduced themselves and communicated the basic rules of the interview. The child was told to report the truth and that it was acceptable to answer “I do not understand” or “I do not know” if a question was not understood. Once the child understood the instructions, the interviewer asked open-ended questions and “yes/no” questions to assess the child’s memory of the play session. The memory interview was hierarchically structured (e.g., Baker-Ward et al., 2015; Boland et al., 2003; Hedrick et al., 2009) so that it started with general questions, followed by specific questions, and finally “yes/no” questions. Accordingly, the child was first asked general open-ended questions that encouraged the child to freely recall their experience about the play session (e.g., “Can you tell me everything that you remember about the play?”). Then, the interviewer asked follow-up questions referring to the child’s freely recalled information (e.g., “Earlier you mentioned the hat. Can you tell me more about that?”) to gain more detailed information. After maximizing the child’s responses to these questions, 20 specific “yes/no” questions were posed: 10 questions concerning what happened (i.e., present features) during the play session and 10 suggestive questions about aspects that were not included in the session (i.e., absent features).

### 3.2 Coding

The memory interviews were audio recorded and transcribed with parents’ consent and children’s assent. Established procedures (Ornstein et al., 2006) were used as guidelines when quantifying children’s recall accuracy. For open-ended questions, we counted the number of recalls based on first mention; items stated repeatedly were not counted more than once. A child’s correct responses to open-ended questions (e.g., “Can you tell me everything that you remember from the play?”) were coded as *free recall.* For “yes/no” questions, affirmative responses to suggestive questions (i.e., questions about an event that had not occurred) were coded as *suggestibility*. The scores used in our final analysis were calculated by dividing the total number of children’s responses by the total number of items for each question type. For example, if the free recall score was 0.20, then a child responded correctly to 4 out of 20 open-ended questions. A suggestibility score of 0.50 indicated that the child incorrectly replied “yes” to 5 of 10 suggestive questions.

### 3.3 Data analysis

All analyses were performed in IBM SPSS for Windows, v. 26.0 (IBM Corp., Armonk, NY, USA). For our preliminary analyses, a *t* test was conducted to identify any significant differences in participants’ memory performance (free recall, suggestibility) depending on gender. Although uncommon, significant gender differences have been reported where girls recalled more information than boys (e.g., Salmon and Pipe, 1997). In our study, gender did not significantly affect children’s free recall and suggestibility; this variable was thus excluded from analysis.

For the main analyses, two-way ANOVAs were performed to compare the effects of age and experimental conditions on children’s free recall and suggestibility. We further implemented follow-up tests (i.e., pairwise comparisons) to clarify interaction effects in the ANOVA results.

## 4 Results

Two-way ANOVAs were carried out to assess the effects of experimental conditions on children’s free recall and suggestibility depending on age. The means, standard deviations, and ANOVA results for children’s free recall and suggestibility according to age and experimental conditions are presented in Tables 1, 2.

We observed significant main effects of age on free recall, *F*(1, 56) = 184.72, *p* < 0.001, partial η^2^ = 0.77, and on suggestibility, *F*(1, 56) = 33.65, *p* < 0.001, partial η^2^ = 0.38. Pairwise comparisons showed that older children (*M* = 0.43, *SD* = 0.08) reported significantly more accurate free recall than younger children (*M* = 0.17, *SD* = 0.08), *p* < 0.001. Older children (*M* = 0.04, *SD* = 0.05) were also significantly more resistant to suggestive information than younger children (*M* = 0.14, *SD* = 0.09), *p* < 0.001.

The main effect of experimental condition was statistically significant for free recall, *F*(1, 56) = 16.24, *p* < 0.001, partial η^2^ = 0.23, and for suggestibility, *F*(1, 56) = 11.95, *p* = 0.001, partial η^2^ = 0.18. The free recall scores of children in the open-ended rapport condition (*M* = 0.34, *SD* = 0.14) were significantly higher than those for children in the NET condition (*M* = 0.26, *SD* = 0.17), *p* < 0.001. Children who experienced open-ended rapport (*M* = 0.06, *SD* = 0.06) demonstrated greater resistance to suggestive information than those who received NET training (*M* = 0.12, *SD* = 0.10), *p* = 0.001.

The interaction effect of age and experimental condition was not significant for free recall, *F*(1, 56) = 2.63, *p* = 0.11, partial η^2^ = 0.05. However, the combined effect of age and experimental condition was significant for suggestibility, *F*(1, 56) = 4.87, *p* = 0.03, partial η^2^ = 0.08.

**Figure 1:**
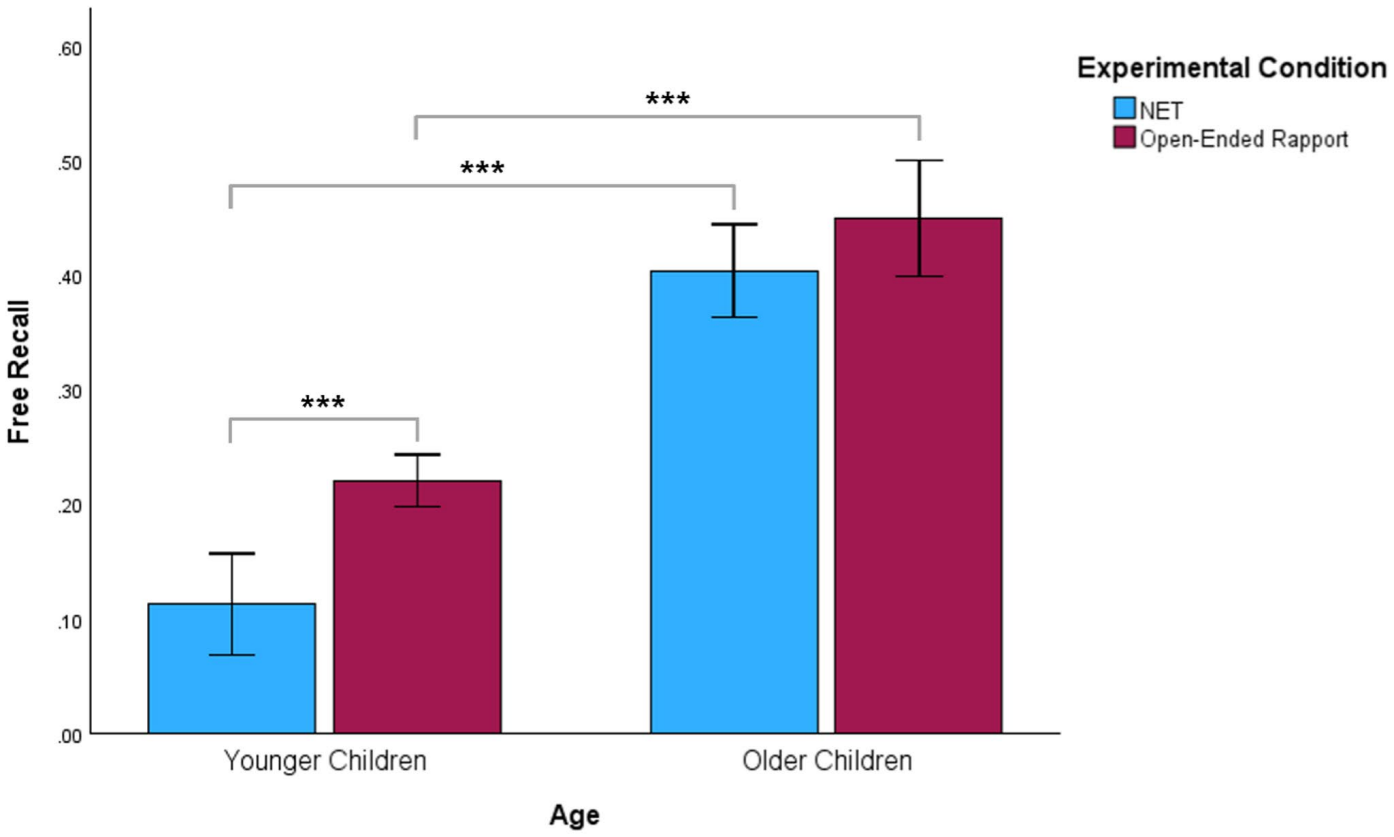
Comparison of the effects of age and experimental conditions on children’s free recall. Free recall scores for NET and open-ended rapport conditions are presented according to age group. Error bars show 95% confidence intervals. ^***^p < 0.001.

We first compared free recall between the two experimental groups for each age group. We observed that younger children in the open-ended rapport condition (*M* = 0.22, *SD* = 0.04) had significantly higher free recall scores than those in the NET condition (*M* = 0.11, *SD* = 0.08), *p* < 0.001. No significant differences emerged between the free recall scores of older children in the open-ended rapport condition (*M* = 0.45, *SD* = 0.09) and older children in the NET condition (*M* = 0.40, *SD* = 0.07), *p* = 0.09. Next, we compared free recall between younger and older children in the same experimental condition. Older children in the open-ended rapport condition (*M* = 0.45, *SD* = 0.09) performed significantly better than younger children in this condition (*M* = 0.22, *SD* = 0.04), *p* < 0.001. Similarly, older children in the NET condition (*M* = 0.40, *SD* = 0.07) recalled information more accurately than younger children in this condition (*M* = 0.11, *SD* = 0.08), *p* < 0.001. Figure 1 illustrates free recall scores for the NET and open-ended rapport conditions according to age group.

**Figure 2:**
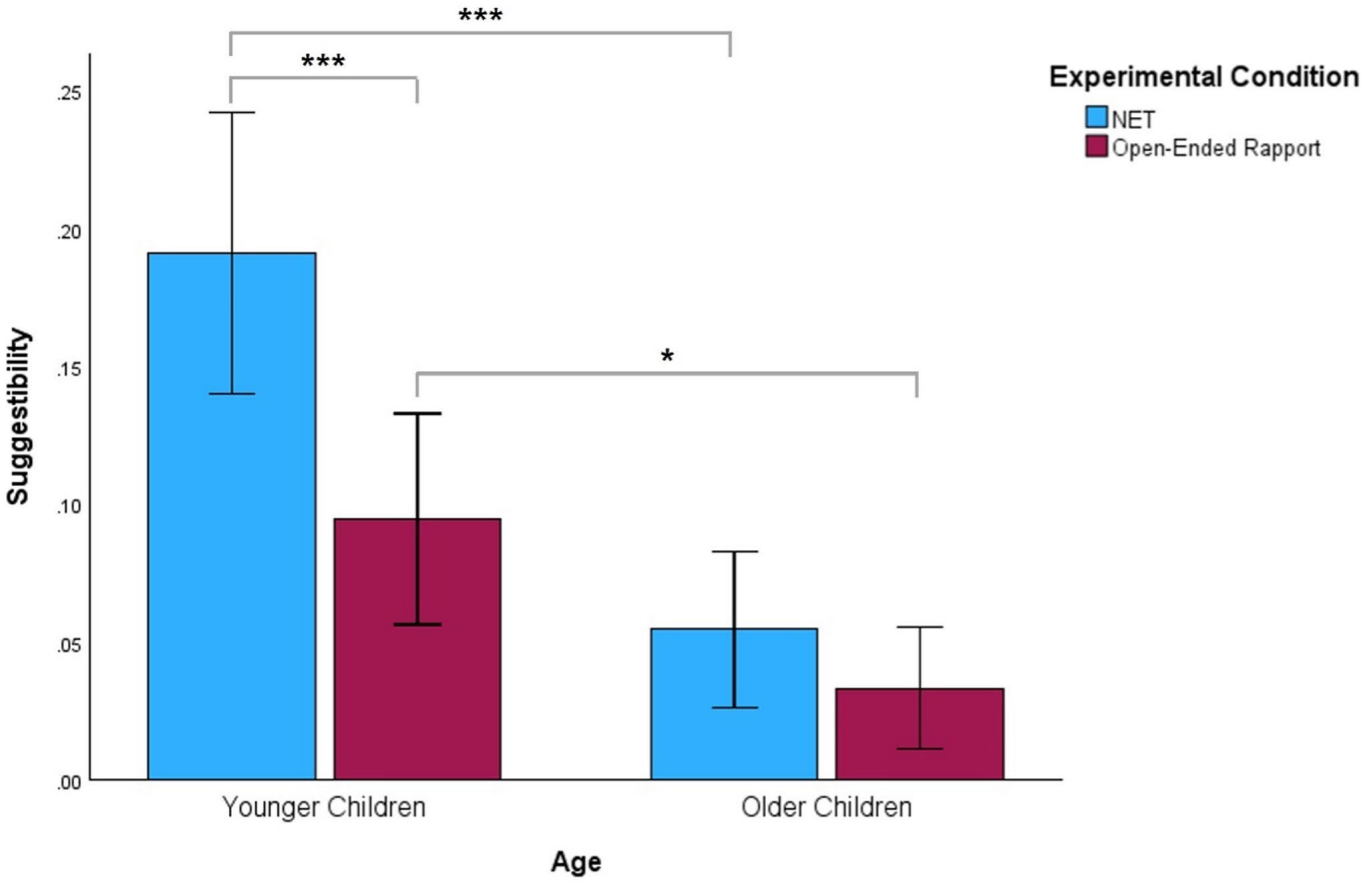
Comparison of the effects of age and experimental conditions on children’s suggestibility. Suggestibility scores for NET and open-ended rapport conditions are presented according to age group. Error bars show 95% confidence intervals. ^*^p < 0.05. ^***^p < 0.001.

A similar pattern emerged when we examined the effects of the experimental conditions on suggestibility by age group. Younger children in the open-ended rapport condition (*M* = 0.09, *SD* = 0.07) were significantly more resistant to suggestive questions than those in the NET condition (*M* = 0.19, *SD* = 0.09), *p* < 0.001. There were no significant differences in the suggestibility scores between older children in the open-ended rapport condition (*M* = 0.03, *SD* = 0.04) and older children in the NET condition (*M* = 0.05, *SD* = 0.05), *p* = 0.38. We examined the suggestibility of younger and older children who were in the same experimental condition as well. Regardless of condition, older children’s suggestibility scores were significantly lower than those of younger children. Specifically, older children in the open-ended rapport condition (*M* = 0.03, *SD* = 0.04) were significantly more resistant to suggestive questions than younger children in this condition (*M* = 0.09, *SD* = 0.07), *p* = 0.01. Older children who received NET (*M* = 0.05, *SD* = 0.05) were more resistant to suggestive questions than younger children who received NET (*M* = 0.19, *SD* = 0.09), *p* < 0.001. Figure 2 depicts differences in suggestibility scores between children who received NET and those who received open-ended rapport according to age group.

In sum, our results suggest that the impact of NET or open-ended rapport on children’s memory performance may vary with age. Free recall and suggestibility did not significantly differ by the experimental condition among older children. On the contrary, younger children’s memory performance was significantly better in the open-ended rapport condition compared with the NET condition. These findings provide implications for the importance of open-ended rapport building in investigative interviews with younger children.

## 5 Discussion

Young children can provide accurate and reliable statements regarding events they have witnessed or experienced (Brainerd and Reyna, 2012; Otgaar et al., 2018). However, younger children often have trouble providing information that is sufficiently robust and specific to inform judicial decisions due to these participants’ immature cognitive and language development (Perez et al., 2022; Turner and Hughes, 2022). Scholars have therefore emphasized the importance of efficiently building rapport and offering additional tools to younger children during investigative interviews (Saywitz et al., 2015; Wolfman et al., 2018).

This study examined the effectiveness of NET and open-ended rapport building on children’s memory according to age. Open-ended rapport had a more positive impact on younger children’s memory when compared to NET. Meanwhile, older children’s memory performance did not significantly differ by interview strategy. These outcomes suggest that it is essential to give thought to the techniques used when interviewing young children forensically (Baugerud et al., 2020). The effectiveness of NET might have been relatively lower with young children because of their cognitive and developmental limitations. Canning and Peterson (2020) demonstrated that NET training significantly enhanced only 5- to 7-year-olds’ recall accuracy; 3- to 5-year-olds did not benefit from this training. Dorado and Saywitz (2001) noticed that NET did not significantly influence young children’s free recall or responses to open-ended “who,” “what,” and “where” questions. NET training also exhibited limited effectiveness for young children: Dorado and Saywitz (2001) reported that young children in the NET condition performed better than the control group only when the NET group was shown visual NET cue cards and its participants were asked to think about whether there was any more information they would like to share. Inconsistent findings regarding NET’s utility with young children implies that these children are limited in their capacity to transfer training to new tasks. Open-ended rapport could have helped younger children feel emotionally stable and secure, leading to higher memory performance (Vallano and Schreiber Compo, 2015).

By contrast, older children’s memory may not have varied with the interview strategies (open-ended rapport or NET training) because these children have relatively more advanced language and cognitive development. Children can structure information beginning at age 7, and these skills enhance memory (Schneider and Pressley, 2013). Children become increasingly capable of efficiently selecting and applying contextually appropriate memory strategies with age (Courage and Cowan, 2022). Our results also indicate that older children demonstrated greater memory performance, were more spontaneous in their memory retrieval, and were less vulnerable to suggestion than younger children. Considering these findings, police officers should consider using open-ended rapport building to help young children provide accurate, complete information during investigative interviews.

## 6 Strengths and limitations of the study

This study contributes to the literature by expanding understanding of the roles of open-ended rapport building and NET training on younger and older children’s memory performance. Many NET studies have focused exclusively on school-age children (e.g., Brown and Pipe, 2003a, 2003b; Larsson and Lamb, 2009; Saywitz and Snyder, 1996). Including young children in our sample enabled us to examine how the interaction between children’s age and interview strategy can influence their memory. Findings suggested that open-ended rapport promotes young children’s memory performance more effectively than NET training. This consequence has notable implications in forensic settings. Our research can serve as a reference for police officers and investigators who are seeking effective strategies for completing investigative interviews with young children. Rather than devoting substantial effort to implementing both open-ended rapport and NET, police officers could conduct more efficient interviews with this population by solely building open-ended rapport prior to an interview.

This study has limitations that can be addressed in future research. First, we did not explore factors that might explain why certain interview tactics are more useful than others in boosting children’s accurate recall (Peterson, 2012). Emotional stability is known to influence children’s performance on cognitive tasks (Blankson et al., 2013). Younger children in the open-ended rapport condition might have performed better than those in the NET condition because the former group was feeling more emotionally stable. Another explanation could be young children’s confidence. Open-ended rapport also aims to increase children’s confidence as information providers. The memory performance of young children in the open-ended rapport condition may be associated with the confidence that they gained from the open-ended rapport experience. Scholars should contemplate potential mediators and moderators of interview strategies to gain clarity around why certain techniques are especially impactful in increasing young children’s memory performance.

Second, we assessed the effectiveness of NET and open-ended rapport building on children’s memory performance without a control group. We cannot say whether NET or open-ended rapport is more beneficial in comparison to interviews that do not feature either tool. However, studies have often demonstrated that children in a NET or open-ended rapport condition were able to report more detailed and accurate information than those in a control group (e.g., Brown and Pipe, 2003a, 2003b; Camparo et al., 2001; Dorado and Saywitz, 2001; Lee, 2024; Roberts et al., 2004). These findings may reinforce our results’ contributions in terms of describing how interview strategies affect young and older children’s memory performance. Future studies can further expand on the effectiveness of NET and open-ended rapport by including a control group.

Finally, we used the same memory interview for all experimental groups to compare the effects of age and experimental condition (open-ended rapport building or NET) on participants’ memory performance. This approach was also adopted in other NET studies to compare the effectiveness of NET and control on participants’ memory recall (e.g., Dorado and Saywitz, 2001; Saywitz et al., 1996). Nonetheless, using the same memory interview for all groups might have partly prevented participants from employing the knowledge and skills they had gained through open-ended rapport or NET. For instance, participants in the NET condition may have shown a greater degree and accuracy of recall if they had been allowed to apply the cues from NET training. For this reason, in future studies, it might be beneficial to conduct the memory interview using a procedure that aligns with each experimental condition.

## Data Availability

The raw data supporting the conclusions of this article will be made available by the authors, without undue reservation.
